# Epigenetic inactivation of *HOXA11*, a novel functional tumor suppressor for renal cell carcinoma, is associated with RCC TNM classification

**DOI:** 10.18632/oncotarget.15668

**Published:** 2017-02-24

**Authors:** Lu Wang, Yun Cui, Jindong Sheng, Yang Yang, Guanyu Kuang, Yu Fan, Jie Jin, Qian Zhang

**Affiliations:** ^1^ Department of Urology, Peking University First Hospital and Institute of Urology, Peking University, Beijing 100034, China; ^2^ Department of Urology, National Research Center for Genitourinary Oncology, Peking University First Hospital, Beijing 100034, China; ^3^ Department of Urology, National Urological Cancer Center, Peking University First Hospital, Beijing 100034, China

**Keywords:** HOXA11, methylation, renal cell carcinoma

## Abstract

Epigenetic inactivation of *HOXA11*, a putative tumor suppressor, is frequently observed in a number of solid tumors, but has not been described in RCC (renal cell carcinoma). In this study, we investigated the expression, epigenetic changes and the function of *HOXA11* in human renal cell carcinoma (RCC). *HOXA11* was silenced or down-regulated in RCC cell lines and tissues. Methylation specific PCR (MSP) and bisulfite genomic sequencing (BGS) revealed that the *HOXA11* promoter was hypermethylated in 5/6 RCC cell lines. Demethylation treatment resulted in demethylation of the promoter and increased *HOXA11* expression in these cell lines. *HOXA11* methylation was also detected in 68/95 (70.5%) primary RCC tumors, but only rare adjacent non-malignant renal tissues (13%, 3/23) showed hypermethylation of promoter. We also found that the methylation of *HOXA11* was associated with higher TNM classification of RCC (*p*<0.05). Ectopic expression of *HOXA11* led to significant inhibition of proliferation, colony formation, migration and invasion abilities and induced RCC cells apoptosis. Moreover, *HOXA11* was found to inhibit Wnt signaling. Thus, our study demonstrated that *HOXA11* function as a tumor suppressor in RCC, while it is frequently silenced by promoter methylation in RCC.

## INTRODUCTION

Renal cell carcinoma represents 2-3% of human malignant neoplasms [1]. The incidence of renal cell carcinoma is increasing by about 2% annually [2]. It is burdened with a high mortality level, poor prognosis and insensitivity to radiotherapy and chemotherapy, thus it is considered one of the most important urological cancers [3, 4]. Therefore, it is critical to reveal the molecular mechanism underlying RCC tumorigenesis. Renal cell carcinoma develops as a result of inheritance which occurs due to the abnormalities in the genetic material as well as changes acquired as the environment impact [5]. The changes of environment correlate closely to the epigenetic, which has been reported to be a feature of various carcinomas including renal cell carcinoma. Over the past few years, aberrant epigenetic changes have been found in renal cell carcinoma [6].

*HOXA11* is a member of *HOX* transcription factors, the *HOX* genes encode transcription factors that play an essential role in embryonic development and differentiation of adult cells [7]. *HOX* genes are also known to be involved in different stages of kidney organogenesis, from the early events in intermediate mesoderm to terminal differentiation of glomerular and tubular epithelia [8]. *HOX* genes consist of four clusters including A, B, C and D on four different chromosomes [9]. The *HOXA* cluster located on chromosome 7p15-7p14.2 consists of 12 genes including *HOXA11* [10]. *HOXA11* plays an important role in regulating cell differentiation and proliferation [11, 12]. The hypermethylation of *HOXA11* promoter region has been reported in various cancers [13–16]. However, the epigenetic alteration and function of *HOXA11* in human renal cell carcinoma has not been explained. Therefore, to investigate the relationship between *HOXA11* methylation and tumor development becomes important to further elucidate the tumorigenesis of RCC.

To gain better insight into the role of *HOXA11* in RCC, we investigated the expression of *HOXA11* in RCC tissues and cell lines and further characterized the *HOXA11* hypermethylation. Thus, we next analyzed the association between clinicopathological parameters and *HOXA11* methylation in RCC tissues. What's more, functional research showed that *HOXA11* inhibited RCC cell proliferation, migration and invasion ability and induced apoptosis. *HOXA11* also inhibited Wnt signaling. Collectively, our data identifies *HOXA11* as a functional tumor suppressor which is frequently methylated in renal cell carcinoma.

## RESULTS

### Epigenetic inactivation of *HOXA11* in RCC cell lines

**Figure 1:**
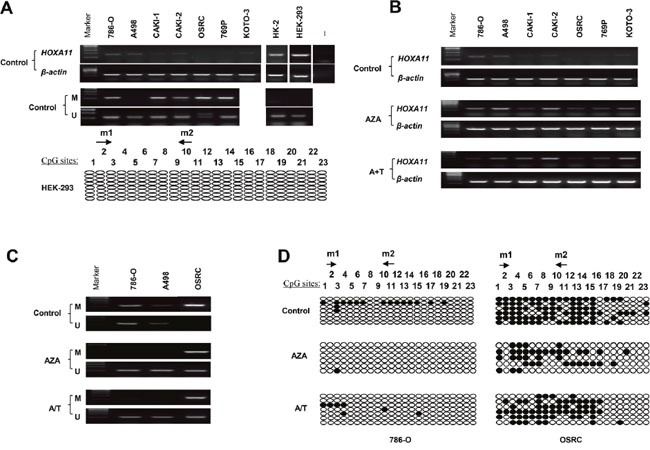
Methylation and expression status of *HOXA11* in RCC cell lines **A.** The mRNA expression and promoter methylation of *HOXA11* was detected by RT-PCR and MSP in RCC cell lines, — represents negative control; BGS analysis of *HOXA11* promoter methylation in HEK-293 cell; **B.** Detection of *HOXA11* expression by RT-PCR after demethylation treatment with Aza or Aza +TSA, A: Aza, T: TSA; **C.** Demethylation treatment induced demethylation in RCC cell lines by MSP, M: mehylation, U: unmethylation; **D.** BGS analysis of *HOXA11* promoter methylation after demethylation treatment, filled circles: methylated CpG site, open circles: unmethylated CpG site.

To examine the expression of *HOXA11*, RT-PCR was employed. *HOXA11* was weakly expressed in 786-O, A498 and CAKI-2, no expression was found in CAKI-1, OSRC, 769P and KOTO-3. However, *HOXA11* is robustly expressed in two approximately “normal” kidney cell lines (HK-2 : “normal” human proximal tubular cell line; HEK-293 : human normal embryonic kidney cell line) (Figure 1A). Aberrant methylation of *HOXA11* promoter was observed in 5/6 RCC cell lines (786-O, A498, CAKI-1, 769P, OSRC and CAKI-2) by MSP. No promoter methylation of *HOXA11* was detected in HEK-293 and HK-2 cells (Figure 1A). To analyze the correlation of *HOXA11* expression and aberrant promoter methylation, RCC cell lines were treated with 5-Aza combined with or without TSA. Enhanced expression of *HOXA11* was shown in 6 RCC cell lines (Figure 1B). In addition, the methylation status of *HOXA11* reduced in 786-O and A498 cells. Even though no reduction of *HOXA11* promoter was observed in OSRC cell, its unmethylated status was up-regulated after demethylation treatment (Figure 1C). The MSP results are consistent with Bisulfite Genomic Sequencing (BGS) results very well (Figure 1D). These results indicate that aberrant methylation of promoter decreased the *HOXA11* expression.

### *HOXA11* was frequently methylated and reduced in human primary RCC samples

**Figure 2:**
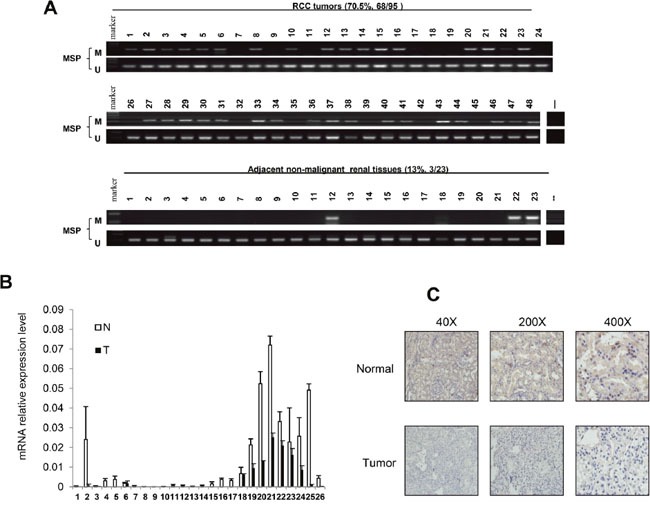
*HOXA11* expression and promoter methylation in primary RCC tissues and adjacent non-malignant renal tissues **A.** MSP analysis of *HOXA11* methylation in primary RCC tissues and adjacent non-malignant renal tissues. **B.** Quantity analysis *HOXA11* mRNA expression level in paired RCC tissues, N: adjacent non-malignant renal tissues, T: primary RCC tissues; **C.** Representative images of *HOXA11* protein expression in RCC tissues and their adjacent non-malignant tissues determined by IHC (immunohistochemistry).

To explore methylation changes of *HOXA11* in RCC tissues and adjacent non-malignant renal tissues, 95 RCC samples and 23 adjacent non-malignant renal tissues were detected by MSP. As Table 1 showed that *HOXA11* was found to be methylated in 70.5% (68/95) of primary RCC samples, while only 13% (3/23) of adjacent non-malignant renal tissues was found to be methylated in *HOXA11* promoter region (Figure 2A). In addition, Real-time PCR was performed in 26 paired RCC tissues and adjacent non-malignant renal tissues. *HOXA11* was reduced in 26/26 RCC tissues compared with adjacent non-malignant renal tissues (Figure 2B). In addition, immunohistochemistry was used to detected the *HOXA11* protein expression in 15 paired RCC tissues and adjacent non-malignant renal tissues, expression of *HOXA11* was decreased in 14/15 (*p*<0.05) RCC tissues (Figure 2C). Collectively, these results further demonstrated that *HOXA11* is frequent down-regulated in tumors with higher methylation status in RCC.

**Table 1:** Methylation status of *HOXA11* in primary RCC tissues and adjacent non-malignant renal tissues

RCC samples	*HOXA10* promoter		Methylation percentage
	Methylation	Unmethylation	
Tumor	68	27	70.5%
Non-malignant	3	20	13%

In addition, we further analyzed the correlation of *HOXA11* methylation and patients’ clinical features. Table 2 listed the clinicopathological features of RCC patients and statistic results. Interestingly, methylation of *HOXA11* was significantly associated with TNM (*p*<0.05). But no association was found with gender, age, side and nuclear grade (*p*<0.05).

**Table 2:** Association of *HOXA11* methylation with clinicopathological features in RCC

Clinicopathological Feature	Methylated NO. (%)		Unmethylated NO. (%)	*p* value
Age		54±12.9	56±12.5	0.63
Gender	Male	44(71%)	18(29%)	0.86
	Female	24(72.7%)	9(27.3%)	
Side	Left	32(71.1%)	13(28.9%)	0.92
	Right	36(72%)	14(28%)	
TNM classification	pT1	41(63.1%)	24(36.9%)	**0.013**
	pT2	6(100%)	0(0%)	
	pT3	18(85.7%)	3(14.3%)	
	pT4	3(100%)	0(0)	
Nuclear grade	G1	5(50%)	5(50%)	0.42
	G2	48(72.7%)	18(27.3%)	
	G3	15(78.9%)	4(22.1%)	

### *HOXA11* suppresses RCC cells proliferation and induces cell apoptosis

**Figure 3:**
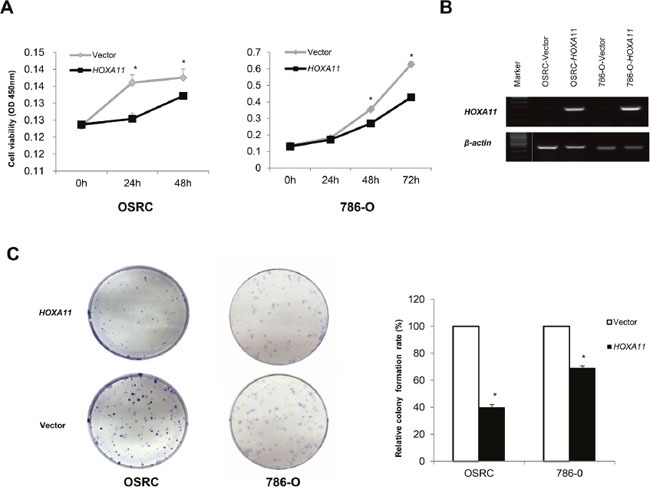
Ectopic expression of *HOXA11* inhibits RCC cell proliferation and colony formation abilities **A.** CCK-8 assay showed an inhibition effect of *HOXA11* on cell growth, *: *p*<0.05; **B.** RT-PCR showed *HOXA11* expression in *HOXA11*- or vector-transfected cells, *β-actin* was used as a control. **C.** *HOXA11* suppressed RCC cells (786-O and OSRC) colony formation, the experiment was repeated for three times as values of mean±SD, *: *p*<0.05.

**Figure 4:**
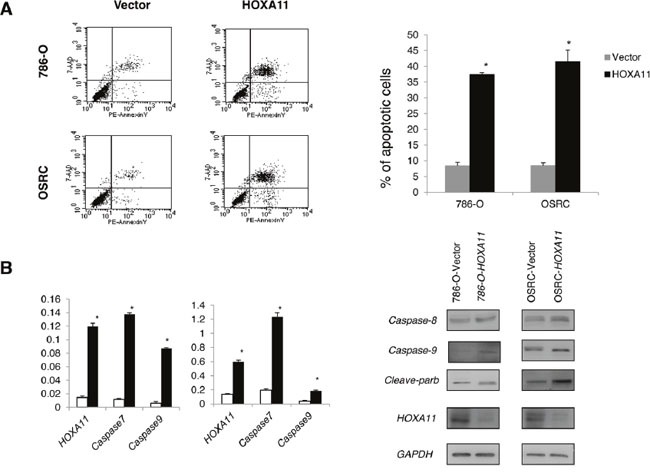
**A.** *HOXA11* induced apoptosis in 786-O and OSRC cells by flow cytometry analysis following Annexin V and 7-AAD staining. Quantitative analyses of apoptotic cells in 786-O and OSRC, *: *p*<0.05. **B.** Effect of *HOXA11* on expression of pro-apoptosis regulators in OSRC and 786-O examined by Real-time PCR and western blot analysis.

The frequent down-regulation and methylation of *HOXA11* in primary RCC tumors indicated that it might function as a tumor suppressor. Thus, we further explored the effects of *HOXA11* in two *HOXA11* deficient RCC cells (786-O, OSRC). CCK8 assay was used to assess the proliferation ability of cells transfected with *HOXA11* and Vector. As it was showed in Figure 3A, *HOXA11* significantly inhibit the proliferation of RCC cells. What's more, results of colony formation assays showed that ectopic expression of *HOXA11* significantly suppressed the numbers of cell colonies compared with the control cell (Figure 3C), indicating that *HOXA11* indeed suppressed the tumorigenesis of RCC. Expression of *HOXA11* was confirmed by RT-PCR (Figure 3B). To evaluate the mechanism of *HOXA11* in cell proliferation, flow cytometric analyses of apoptosis was performed. We found that ectopic *HOXA11* expression in 786-O and OSRC cells underwent significant apoptosis compared to controls (Figure 4A). Then, Real-time PCR and western-blot showed that ectopic expression of HOXA11 repressed the expression of Caspase7, Caspase8, Caspase9 and Cleaved parb. These results indicated that *HOXA11* could suppress the RCC cells proliferation and induce apoptosis.

### *HOXA11* inhibits RCC cell invasion and migration

**Figure 5:**
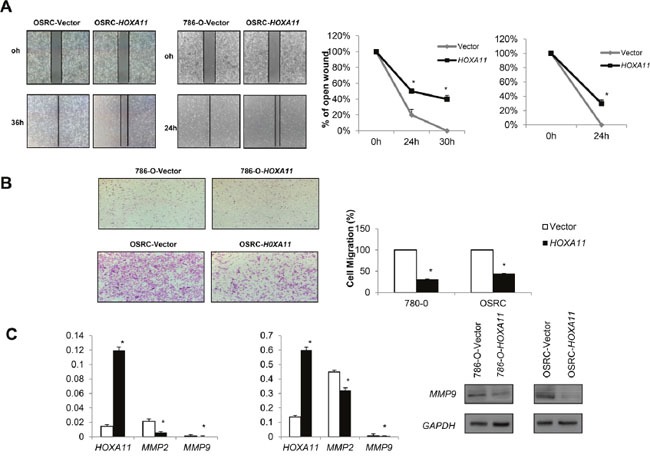
Effects of *HOXA11* on RCC cell migration and invasion **A.** Ectopic expression of *HOXA11* inhibit cell migration ability in 786-O and OSRC, pictures of wound-healing were captured at 0 h, 24 h and 30 h, *p*<0.05; **B.** Transwell assay shows migration results in *HOXA11* unexpressed and re-expressed in RCC cells, bar graphs represent the numbers of migrating 786-O and OSRC cells, the experiment was repeated three times, *: *p*<0.05. **C.** Ectopic expression of *HOXA11* inhibited migration related genes expression in OSRC and 786-O cells examined by qPCR and Western-blot.

Wound-healing and Transwell assay were performed to explore the function of *HOXA11* in RCC cells. As shown in Figure 5A, the wound-healing assay results showed that *HOXA11* expressing cells were less proficient in closing an artificial wound than the empty vector tansfected cells on the confluent monolayer (*p*<0.05). In the Transwell assay, the number of migrated cells transfected with *HOXA11* of each field was less compared with the control cells (Figure 5B, *p*<0.05). What's more, *HOXA11* decreased *MMP9* and *MMP2* expression, protein that promoted cell migration. Above results hint that *HOXA11* suppresses the ability of invasion and migration in RCC cells.

### Effect of *HOXA11* on Wnt/β-catenin signaling

**Figure 6:**
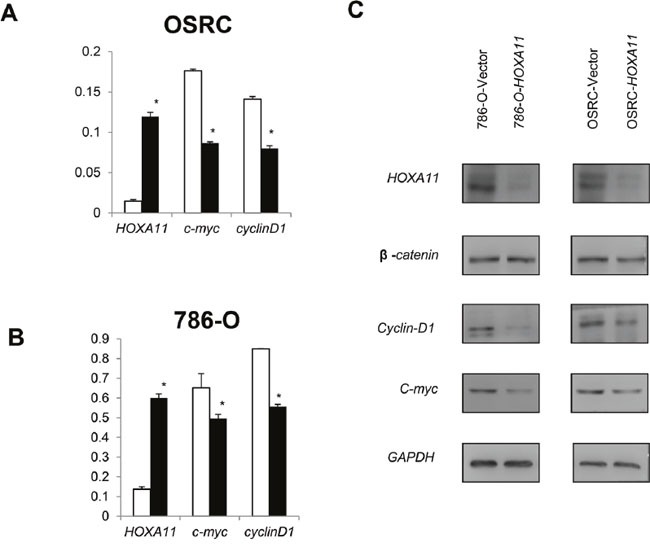
Effect of *HOXA11* on Wnt signaling down-stream target genes expression **A, B.** In OSRC and 786-O cells, over-expression of *HOXA11* reduced *c-myc* and *cyclinD1* expression, values are presented as mean±SD, *p*<0.05.

Canonical Wnt signaling pathway plays a significant role in RCC tumorigenesis. To explore the effect of *HOXA11* on Wnt signaling pathway in RCC, Real-time PCR was employed. As the Figure 6A, 6B and 6C showed, the down-stream genes (*c-myc*, *cyclinD1*) of Wnt signaling pathway was apparently reduced in *HOXA11* expressed cells compared with control cells. These results suggested that *HOXA11* might function as antagonist of Wnt/β-catenin signaling pathway.

## DISCUSSION

In this study, we found that *HOXA11* was expressed broadly in human normal kidney tissues. However, it is frequently methylated and decreased in RCC tissues. Pharmacologic demethylation resulted in the demethylation and restoration of *HOXA11* expression. We also found that *HOXA11* functions as a tumor suppressor through antagonizing WNT/βcatenin signaling and inhibits RCC cells proliferation, migration and invasion. Our study demonstrated the role of *HOXA11* in RCC and its regulation mechanism, and strongly supports the notion that *HOXA11* is a tumor suppressor for multiple carcinomas.

*HOX* genes encode a DNA binding motif and regulate gene transcription [17]. However, the mechanisms of *HOX* genes in tumorigenesis have not been elucidated. *HOXA11*, one member of *HOX* genes, is often deleted in human cancers [18, 19]. The deletion of *HOXA11* has been reported to cause a specific syndrome with skeletal defects and amegakaryocytic thrombocytopenia [20]. Hypermethylation and low expression of *HOXA11* was reported in different cancers, such as ovarian cancer and endometrial cancer, and it functions as a tumor suppressor [13, 21]. In breast cancer, *HOXA11* has been proposed as a biomarker that could be used for early detection [22]. In ovarian cancer, the methylation of *HOXA11* has been reported to be a poor prognostic marker [13]. Demthylation treatment with decitabine restored *HOXA11* sensitivity to platinum in patients with platinum-resistant ovarian cancer in a Phase II clinical trial [21]. In addition, the down-regulation of *HOXA11* contributes to the loss of tumor suppressive function in gastric cancer (GC). Cui *et* al. reported that *HOXA11* was frequently methylated in human gastric cancer, ectopic expression of *HOXA11* antagonized WNT/βcatenin signaling [22]. Also, in non-small lung cancer (NSCLC), hypermethylation of *HOXA11* was reported to promote the progression of NSCLC by increasing the proliferation and migration [15]. These previous studies were associated with our results that *HOXA11* was more likely to be methylated in RCC tissues than adjacent non-malignant renal tissues. Over-expression of *HOXA11* inhibits RCC progression through suppressed WNT/βcatenin signaling. However, the detailed mechanism of *HOXA11* tumor suppressive role needs more exploration.

It is currently believed that genetic and epigenetic events could interact to result in tumorigenesis [23]. To combine both genetic and epigenetic may provide a new viewpoint to further understand the pathogenesis of renal cell carcinoma. In addition to genetic changes, tumors can also be considered as an epigenetic disease, including DNA methylation, histone modification and RNA interference [24]. Recently, great attention has been aroused on the significance of DNA methylation for early diagnosis and prognosis prediction in RCC. DLEC1, a tumor suppressor in prostate, has been reported to be methylated in prostate. DLEC1 methylation was associated with higher PSA levels, higher Gleason score, and more advanced tumor stage, which could be a non-invasive epigenetic biomarker for prostate cancer [25]. DNA methylation includes hypermethylation of region rich in cytosine and guanine dinucleotides (CpG islands, CpGIs) within the promoter that results in the inactivation of tumor suppressor genes (TSG) [26]. It has been reported that numerous genes (function as tumor suppressors) were hypermethylated in primary RCC, such as *CDH1*, *APAF1*, *PCDH17*, *SFRP1*, *SFRP4* and *TCF21*. However, these genes are not methylated or rarely methylated in matched normal renal tissues [27–29]. These genes participated in different aspects of tumorigenesis, including apoptosis, signal transduction, angiogenesis and tumor invasion. Another study reported that 55 genes of about 14,000 were methylated in ccRCC but not in normal controls in 38 ccRCCs and 9 matched normal kidney tissues [30]. Genome-scale methylation analysis is a potential tool to identify multiple gene profiles reflecting tumor behavior. Aberrant methylation of *HOXA11* has been reported in several tumors [13, 21]. But the mechanism and roles involved in RCC have not been elucidated. Therefore, to further investigate the role of *HOXA11* methylation in RCC development and identify its regulators are necessary. In our study, we found that methylation of *HOXA11* was at a higher percentage in RCC tumors. Although no correlation was found between *HOXA11* methylation and clinical features such as age, gender and nuclear grade, *HOXA11* methylation was associated with TNM classification. It was known that tumor TNM stage was another independent predictor for overall survival. Thus, *HOXA11* might be used as predictor for clinical survival.

In summary, our study identifies *HOXA11* as a functional tumor suppressor and an important regulator of WNT/βcatenin signaling, with frequent epigenetic inactivation in RCC tumors. Our study further explained the role of *HOXA11* in RCC tumorigenesis.

## MATERIALS AND METHODS

### Primary RCC samples

RCC samples and adjacent non-malignant renal tissues were included in this study. The samples were collected as fresh frozen tissue from Urology Department of Peking University First Hospital, Beijing, China. All samples were collected with the patients’ informed consent. The application of these samples was approved by the hospital ethics committees. All cases were collected from primary surgical resection with no adjuvant therapy. The pathological diagnosis was confirmed at the Pathology Department, Institute of Urology, Peking University First Hospital. The classification of tumor histopathology was on the basis of 2002 AJCC TNM stage and a Fuhrman nuclear grade.

### Cell culture and demethylation treatment

Seven RCC cell lines (786-O, A498, CAKI-1, CAKI-2, OSRC, 769P, KOTO-3) and two approximately “normal” kidney cell lines (HK-2 : “normal” human proximal tubular cell line; HEK-293 : human normal embryonic kidney cell line) were included in our study. The RCC cell lines were originally obtained from American Type Culture Collection, VA, USA. These cell lines were maintained in RPMI1640 or DMEM medium (Invitrogen, CA, USA), supplemented with 10% fetal bovine serum (FBS) (Invitrogen, Carlsbad, CA), 1% penicillin G and 1% streptomycin at 37 °C in humidified CO _2_ (5%) incubator. RCC cells were split to a low density (30% confluence) for 12 h before drug treatment, then treated with 5-aza-2′-deoxycytidine (5-Aza; Sigma®, Hong Kong, China) at a concentration of 10 μM in the optional medium, which was exchanged every 24 h for 72 h, then RCC cells were further treated with 100 nmol/L histone deacetylase inhibitor TSA (trichostatin A) and 10 μM 5-Aza for additional 24 hours. In the end, RNA/DNA was isolated as described below.

### DNA/RNA extraction and bisulfite modification

Total RNA of primary RCC tissues and cells was isolated by Trizol reagent (Invitrogen, Carlsbad, USA) according to the instruction. Genomic DNA of primary RCC tissues and cells were extracted according to the manufacturer's instruction supplied by TIANamp Genomic DNA Kits (TIANGEN®, Shanghai, China). Then the DNA sodium bisulfite modification of genomic DNA was carried out with EpiTect Bisulfite Kit (Qiagen, Hilded, Germany) as the instruction described.

### Semiquantitative RT-PCR and real-time PCR

RNA quality and quantity analysis was evaluated using Agrarose gel electrophoresis with GoTaq® Green Master Mix (Promega, Madison, WI, USA) and spectrophotometry (ABI Prism 7500™ instrument, Applied Biosystems) with SYBR Green PCR Mix. First strand cDNA was synthesized with the TransScriptR First-Strand cDNA Synthesis SuperMix (TransGen Biotech, Beijing). 1 μl cDNA was used for 12.5 μl PCR reaction. PCR primers and corresponding conditions were supplied in the Supplementary Table 1. *β-actin* was used as an internal control.

### MSP and BGS

MSP (Methylation Specific PCR) reaction incorporated bisulfite-treated DNA, RNA enzyme-free H_2_O and GoTaq® Green Master Mix (Promega, Madison, WI, USA) in a final reaction volume of 12.5μl. MSP/USP products were analyzed using 2% agarose gel electrophoresis. The cycle condition and primers were presented in the Supplementary Table 1. The products of bisulfite-treated DNA were cloned into the pEASY-T5 zero vectors (TransGen Biotech Co. Ltd., Beijing), seven to eight clonies were randomly chosen and sequenced by BGS (Bisulfite Genomic Sequencing).

### *HOXA11* expression plasmid construction

The vector containing human full-length *HOXA11* cDNA and the nagtive-control vector were obtained from YouBio company (Hunan, China). The sequence of the *HOXA11* cDNA region was confirmed by the PCR and DNA sequencing. 786-O and OSRC cells were transfected with PLVX-IRES-ZS-*HOXA11* and empty vector using LipofectamineR 3000 Transfection Reagent (Invitrogen, USA) according to the manufacture's instruction.

### Colony formation & cell proliferation detection

Cells were transfected with PLVX-IRES-ZS-*HOXA11* and empty vector using Lip 3000. After 24 h of transfection, cells were seeded at a density of 400, 800, 1200 cells/well in 6-well plates to grow for 10-14 days with G418 (0.4 mg/ml). Then cells were fixed with 75% ethanol for 30 minutes, stained with 0.2% crystal violet (Beyotime, Nanjing, China) for 40 min and then colonies (>50 cells/clony) were counted. Cells were plated in 96-well plate at a density of 2000 cells/well, and cell proliferation ability was measured at 0, 24, 48, 72 h using CCK-8 assay (Dojindo, Kumamoto, Japan). Absorbance was at a wavelength of 450 nm.

### Wound-healing and transwell assay

RCC cells were grown to confluent monolayers on 6-well plates and carefully wounded using sterile tips, then washed twice with fresh medium. After incubation for 12 and 24 h, the wound images were taken with a microscope, then wound gap widths were measured. For Transwell assay, RCC cells were added to the upper chamber with a 24-well Transwell inserts (8μm pore filters, BD Biosciences, Bedford, MA) at a density of 2×10^4^ cells/chamber without serum. Then, medium with 10% FBS was placed in the lower well and incubated overnight followed by removal of cells remained in on the top chamber with cotton swabs. Finally, cells penetrated to the lower membrane surface were fixed in 4% paraformaldehyde, stained with crystal violet and counted in three independent high-power fields (x200). The experiments were performed in triplicate.

### Annexin V apoptosis assay

The RCC cells transfected with *HOXA11*- expressing vectors or empty vectors were harvested after transfected for 48 h. Then the cells were stained with Annexin V (FITC-conjugated) and 7-AAD (BD Biosciences, Bedford, MA) and then sorted by Becton Dickinson LSRII (BD Biosciences, Bedford, MA) according to the manufacturer's protocol.

### Western –blot analysis

Cells were plated 24 h before transfection, cells were harvested 48 h after transfection and total protein was extracted by KeyGEN BioTECH protein extraction kit (KGP1100). For western-blot analysis, protein were separated on 10% SDS-PAGE and transferred onto nitrocellulose membrane. After blocking with 5% fat-free milk for three hours at room temperature, Blots were immunostained with primary antibodies and secondary antibodies respectively. The antibodies were as follows: Caspase 8 (D35G2); Caspase 9 (9502); cleaved-parb (5625); HOXA11 (Abcam, England); GAPDH (Transgen BIOTECH, China); MMP9 (3852S, CST); β-catenin (8480P, CST); Cyclin-D1 (2926P, CST); C-myc (5605P, CST).

### Statistical analysis

The results are expressed as mean±standard deviation (SD) and SAS 9.0 software was employed. The statistical analysis was performed using the Fisher's exact test, Student's t test and Chi-square test. *p* < 0.05 was considered as statistically significant.

## SUPPLEMENTARY MATERIALS TABLE


