# Supplementary material for: Epigenetic inactivation of HOXA11, a novel functional tumor suppressor for renal cell carcinoma, is associated with RCC TNM classification
Source: Oncotarget. 2017 Feb 24;8(13):21861–70. doi: 10.18632/oncotarget.15668 (PMC5400629; doi:10.18632/oncotarget.15668)
Supplement: Supplementary file 1 [file oncotarget-08-21861-s001.pdf]

Epigenetic inactivation of *HOXA11*, a novel functional tumor suppressor for renal cell carcinoma, is associated with RCC TNM classification

SUPPLEMENTARY MATERIAL

Supplementary Table 1: Primers for PCR, MSP and BGS

Primer Name	Sequence	Size	Tm
β-actin-R	TCCTGTGGCATCCACGAAACT	315	61.9
β-actin-F	GAAGCATTTGCGGTGGACGAT		61.8
HOXA11-F	CAGCAGAGGAGAAAGAGCGG	152	59.4
HOXA11-R	CAGCTCTCGGATCTGGTACT		60.1
HOXA11-MF	TTTTAGGTTTAATTCGTGCGC	113	60.1
HOXA11-MR	ACGAACTCCTCCGCGAAATA		59.5
HOXA11-UF	TTTAGGTTTAATTTGTGTGT	112	54.4
HOXA11-UR	ACAAACTCCTCCACAAAATA		53.8
HOXA11-BF	TTAATTTGTTTTAGGTTTAAT	274	50
HOXA11-BR	AAAACCTATAACAAAAC		48.6
MMP-9F	GGGACGCAGACATCGTCATC	139	60.87
MMP-9R	TCGTCATCGTCGAAATGGGC		60.8
MMP-2F	GATACCCCTTTGACGGTAAGGA	112	59.23
MMP-2R	CCTTCTCCCAAGGTCCATAGC		59.86
c-myc-F	GTCAAGAGGCGAACACACAAC	162	60.00
c-myc-R	TTGGACGGACAGGATGTATGC		60.13
cyclinD1-F	GCTGCGAAGTGGAAACCATC	135	59.83
cyclinD1-R	CCTCCTTCTGCACACATTTGAA		59.11
Caspase7-F	CGGTCCTCGTTTGTACCGTC	206	60.73
Caspase7-R	CGCCCATACCTGTCACTTTATCA		60.43
Caspase9-F	CTCAGACCAGAGATTCGCAAAC	116	59.33
Caspase9-R	GCATTTCCCCTCAAACTCTCAA		58.84
